# A Note on the Drazin Indices of Square Matrices

**DOI:** 10.1155/2014/361349

**Published:** 2014-02-10

**Authors:** Lijun Yu, Tianyi Bu, Jiang Zhou

**Affiliations:** ^1^College of Automation, Harbin Engineering University, Harbin 150001, China; ^2^College of Science, Harbin Engineering University, Harbin 150001, China; ^3^College of Computer Science and Technology, Harbin Engineering University, Harbin 150001, China

## Abstract

For a square matrix *A*, the smallest nonnegative integer *k* such that rank (*A* ^*k*^) = rank (A^k+1^) is called the Drazin index of *A*. In this paper, we give some results on the Drazin indices of sum and product of square matrices.

## 1. Introduction

For *A* ∈ *ℂ* ^*n*×*n*^, the smallest nonnegative integer *k* such that rank⁡(*A* ^*k*^) = rank⁡(*A* ^*k*+1^) is called the *Drazin index* of *A*, denoted by ind⁡(*A*). The *Drazin inverse* of *A* is the unique matrix *A* ^*D*^ satisfying *A* ^*k*+1^ *A* ^*D*^ = *A* ^*k*^,  *A* ^*D*^ *AA* ^*D*^ = *A* ^*D*^, and *AA* ^*D*^ = *A* ^*D*^ *A*, where *k* = ind(*A*) (see [1–3]). When ind⁡(*A*) ⩽ 1, *A* ^*D*^ is called the *group inverse* of *A*, denoted by *A* ^#^. If ind⁡(*A*) = 0, then *A* ^*D*^ = *A* ^#^ = *A* ^−1^. The theory of generalized inverses is an active research field in computational mathematics, and the Drazin index plays an important role in the study of the Drazin (group) inverse. Some results on the Drazin indices of matrices (operators) can be found in [4–9].

For any *A* ∈ *ℂ* ^*n*×*n*^, it is known [10] that there exist nonsingular matrices *P*, Δ and nilpotent matrix *N* such that $A=P\left(\begin{array}{cc} \Delta & 0\\ 0 & N \end{array}\right)P^{-1}$. In this case, $A^{D}=P\left(\begin{array}{cc} \Delta^{-1} & 0\\ 0 & 0 \end{array}\right)P^{-1}$, and ind⁡(*A*) is the smallest nonnegative integer *k* such that *N* ^*k*^ = 0; that is, ind⁡(*A*) = ind⁡(*N*). Hence, ind⁡(*A*) is the smallest nonnegative integer *k* such that the group inverse of *A* ^*k*^ exists.

In this paper, we give some results on the Drazin indices of sum and product of square matrices.

## 2. Some Lemmas

In order to prove our main results, we give some lemmas as follows.

**Lemma 1 (see [7])**

For any *A* ∈ *ℂ* ^*n*×*n*^ and nonnegative integer *p*, the limit  lim⁡_*ε*→0^+^_ *ε* ^*p*^(*A* + *εI*)^−1^ exists if and only if *p*⩾*ind*(*A*).

**Lemma 2 (see [8, 11])**

Let $M=\left(\begin{array}{cc} A & B\\ 0 & C \end{array}\right)$ be a square complex matrix, where *A* is square. Then,

(1) $$\begin{array}{c} \max\left\{ind\left(A\right),ind\left(C\right)\right\}⩽ind\left(M\right)⩽ind\left(A\right)+ind\left(C\right). \end{array}$$

**Lemma 3 (see [8, 11])**

Let $M=\left(\begin{array}{cc} A & 0\\ B & C \end{array}\right)$ be a square complex matrix, where *A* is square. Then,

(2) $$\begin{array}{c} \max\left\{ind\left(A\right),ind\left(C\right)\right\}⩽ind\left(M\right)⩽ind\left(A\right)+ind\left(C\right). \end{array}$$

**Lemma 4 (see [12])**

Let $M=\left(\begin{array}{cc} A & B\\ 0 & 0 \end{array}\right)$ be a square complex matrix, where *A* is square. Then, *M* ^#^ exists if and only if *A* ^#^ exists and rank⁡(*M*) = rank⁡(*A*).

**Lemma 5 (see [12])**

Let $M=\left(\begin{array}{cc} A & 0\\ B & C \end{array}\right)$ be a square complex matrix, where *A* is square. Then, *M* ^#^ exists if and only if *A* ^#^, *C* ^#^ exist and rank⁡(*M*) = rank⁡(*A*) + rank⁡(*C*).

**Lemma 6 (see [9])**

For any *A* ∈ *ℂ* ^*m*×*n*^,  *B* ∈ *ℂ* ^*n*×*m*^, |*ind*(*AB*) − *ind*(*BA*)|⩽1.

## 3. Main Results

For a square matrix *A*, let *A* ^*π*^ = *I* − *AA* ^*D*^. We first give an upper bound for the Drazin index of the sum of two square matrices.

**Theorem 7**

Let *P*, *Q* be square complex matrices such that *P* ^*D*^ *Q* = 0 and *PQP* ^*π*^ = 0. Then,

(3) $$\begin{array}{c} ind\left(P+Q\right)⩽ind\left(P\right)+ind\left(Q\right)-1. \end{array}$$

**Proof**

There exist nonsingular matrices *U*, Δ and nilpotent matrix *N* such that $P=U\left(\begin{array}{cc} \Delta & 0\\ 0 & N \end{array}\right)U^{-1}$. Then, $P^{D}=U\left(\begin{array}{cc} \Delta^{-1} & 0\\ 0 & 0 \end{array}\right)U^{-1}$ and ind(*P*) = ind(*N*). Suppose that $Q=U\left(\begin{array}{cc} Q_{1} & Q_{2}\\ Q_{3} & Q_{4} \end{array}\right)U^{-1}$, where *Q*_1_ is a square matrix with the same order as Δ. By *P* ^*D*^ *Q* = 0 we get *Q*_1_ = 0, *Q*_2_ = 0. Then, $Q=U\left(\begin{array}{cc} 0 & 0\\ Q_{3} & Q_{4} \end{array}\right)U^{-1}$. By *PQP* ^*π*^ = 0 we get *NQ*_4_ = 0.

Note that ind(*P* + *Q*) is the smallest nonnegative integer *k* such that the group inverse of (*P* + *Q*)^*k*^ exists. Since $P+Q=U\left(\begin{array}{cc} \Delta & 0\\ Q_{3} & N+Q_{4} \end{array}\right)U^{-1}$, by Lemma 5, we have ind(*P* + *Q*) = ind(*N* + *Q*_4_). Since *NQ*_4_ = 0, we have *ε*(*N* + *Q*_4_ + *εI*) = (*N* + *εI*)(*Q*_4_ + *εI*). Let *m* = ind(*Q*_4_) + ind(*N*) − 1. By Lemma 1, the limit

(4) $$\begin{array}{c} \underset{\varepsilon \to 0^{+}}{\lim}\varepsilon^{m}{\left(N+Q_{4}+\varepsilon I\right)}^{-1}=\underset{\varepsilon \to 0^{+}}{\lim}\varepsilon^{m+1}{\left(Q_{4}+\varepsilon I\right)}^{-1}{\left(N+\varepsilon I\right)}^{-1} \end{array}$$

exists. So ind(*P* + *Q*) = ind(*N* + *Q*_4_) ⩽ *m* = ind(*Q*_4_) + ind(*N*) − 1. By Lemma 3, we get ind(*Q*_4_) ⩽ ind(*Q*). Since ind(*P*) = ind(*N*), we have ind(*P* + *Q*) ⩽ ind(*P*) + ind(*Q*) − 1.

Now we give an example to show that the upper bound in Theorem 7 can be attained.

**Example 8**

Let $P=\left(\begin{array}{cccc} 1 & -1 & 1 & 0\\ 0 & 0 & 0 & 2\\ 0 & 0 & 0 & 1\\ 0 & 0 & 0 & 0 \end{array}\right)$, $Q=\left(\begin{array}{cccc} 1 & -1 & 2 & -2\\ 1 & -1 & 2 & -2\\ 1 & -1 & 1 & -1\\ 1 & -1 & 1 & -1 \end{array}\right)$. Then, ind(*P*) = ind(*Q*) = 2 and $P^{D}=\left(\begin{array}{cccc} 1 & -1 & 1 & -1\\ 0 & 0 & 0 & 0\\ 0 & 0 & 0 & 0\\ 0 & 0 & 0 & 0 \end{array}\right)$. By computation, we have *P* ^*D*^ *Q* = 0 and *PQP* ^*π*^ = 0. The Drazin index of $P+Q=\left(\begin{array}{cccc} 2 & -2 & 3 & -2\\ 1 & -1 & 2 & 0\\ 1 & -1 & 1 & 0\\ 1 & -1 & 1 & -1 \end{array}\right)$ is 3. So ind(*P* + *Q*) = ind(*P*) + ind(*Q*) − 1.

We can obtain the following result from Theorem 7.

**Corollary 9**

Let *P*, *Q* be square complex matrices such that *PQ* = 0. Then,

(5) $$\begin{array}{c} ind\left(P+Q\right)⩽ind\left(P\right)+ind\left(Q\right)-1. \end{array}$$

It is known that $\text{ind}\left(\begin{array}{cc} A & B\\ C & D \end{array}\right)⩽\text{ind}(A)+\text{ind}(D)+2$ under the conditions *CA* = 0, *CB* = 0 or *BC* = 0, *DC* = 0 (see [13]). A better upper bound is given as follows.

**Theorem 10**

Let $M=\left(\begin{array}{cc} A & B\\ C & D \end{array}\right)$ be a square complex matrix, where *A* is square. Then, *ind*(*M*) ⩽ *ind*(*A*) + *ind*(*D*) + 1 if one of the following holds:

1. *BC* = 0, *DC* = 0;
2. *BC* = 0, *BD* = 0;
3. *CA* = 0, *CB* = 0;
4. *AB* = 0, *CB* = 0.

**Proof**

We only prove part (1). Parts (2)–(4) can be obtained in the same way.

Let $P=\left(\begin{array}{cc} A & B\\ 0 & D \end{array}\right)$, $Q=\left(\begin{array}{cc} 0 & 0\\ C & 0 \end{array}\right)$; then, *M* = *P* + *Q*. By *BC* = 0, *DC* = 0, we get *PQ* = 0. By Corollary 9, we have

(6) $$\begin{array}{c} \text{ind}\left(M\right)⩽\text{ind}\left(P\right)+\text{ind}\left(Q\right)-1⩽\text{ind}\left(P\right)+1. \end{array}$$

By Lemma 2, we have   

(7) $$\begin{array}{c} \text{ind}\left(M\right)⩽\text{ind}\left(A\right)+\text{ind}\left(D\right)+1. \end{array}$$

**Theorem 11**

For *A*, *B* ∈ *ℂ* ^*n*×*n*^, if *s*⩾max⁡{*ind*(*AB*), *ind*(*BA*)}, then (*AB*)^*s*^ and (*BA*)^*s*^ are similar.

**Proof**

Clearly (*AB*)^0^ and (*BA*)^0^ are similar. So we only consider the case *s*⩾1. There exist nonsingular matrices *P*, *Q* such that $A=P\left(\begin{array}{cc} I_{r} & 0\\ 0 & 0 \end{array}\right)Q$, where *I*_*r*_ is the identity matrix of order *r* = rank⁡(*A*). Suppose that $B=Q^{-1}\left(\begin{array}{cc} B_{1} & B_{2}\\ B_{3} & B_{4} \end{array}\right)P^{-1}$, where *B*_1_ ∈ *ℂ* ^*r*×*r*^. Then,

(8) $$\begin{array}{c} AB=P\left(\begin{array}{cc} B_{1} & B_{2}\\ 0 & 0 \end{array}\right)P^{-1},\;\;{\left(AB\right)}^{s}=P\left(\begin{array}{cc} B_{1}^{s} & B_{1}^{s-1}B_{2}\\ 0 & 0 \end{array}\right)P^{-1},\\ BA=Q^{-1}\left(\begin{array}{cc} B_{1} & 0\\ B_{3} & 0 \end{array}\right)Q,\;\;{\left(BA\right)}^{s}=Q^{-1}\left(\begin{array}{cc} B_{1}^{s} & 0\\ B_{3}B_{1}^{s-1} & 0 \end{array}\right)Q. \end{array}$$

Since *s*⩾max⁡{ind(*AB*), ind(*BA*)}, the group inverses of (*AB*)^*s*^ and (*BA*)^*s*^ both exist. Lemmas 4 and 5 imply that there exist matrices *X*, *Y* such that *B*_1_^*s*^ *X* = *B*_1_^*s*−1^ *B*_2_ and *YB*_1_^*s*^ = *B*_3_ *B*_1_^*s*−1^. Then,

(9) (AB)s=P(B1sB1sX00)P−1=P(I−X0I)(B1s000)(IX0I)P−1,(BA)s=Q−1(B1s0YB1s0)Q=Q−1(I0YI)(B1s000)(I0−YI)Q.

Hence, (*AB*)^*s*^ and (*BA*)^*s*^ are similar.

**Corollary 12**

For *A*, *B* ∈ *ℂ* ^*n*×*n*^, if *ind*(*AB*) = *s*, then (*AB*)^*l*^ and (*BA*)^*l*^ are similar for any *l*⩾*s* + 1.

**Proof**

By Lemma 6 and Theorem 11, (*AB*)^*l*^ and (*BA*)^*l*^ are similar for any *l*⩾*s* + 1.

The following corollary is a special case of Theorem 11.

**Corollary 13**

For *A*, *B* ∈ *ℂ* ^*n*×*n*^, if (*AB*)^#^ and (*BA*)^#^ both exist, then *AB* and *BA* are similar.
